# A unique finding of Henoch-Schönlein purpura in adults

**DOI:** 10.11604/pamj.2024.48.59.43884

**Published:** 2024-06-13

**Authors:** Jay Dinesh Bhanushali

**Affiliations:** 1 Department of Respiratory Medicine, Jawaharlal Nehru Medical College, Datta Meghe Institute of Higher Education and Research, Sawangi (Meghe), Wardha, Maharashtra, India

**Keywords:** Vasculitis, hematuria, abdominal pain, arthralgia

## Image in medicine

A 27-year-old male presented in the clinic with a two-day history of sudden onset abdominal pain, vomiting, joint pain, and a rash over his lower limbs. The patient had recently returned from his village and had a history of a recent upper respiratory tract infection. He did not have any significant history. On examination, he had per abdominal tenderness with no guarding rigidity or mass, and bowel sounds were present. The laboratory blood findings showed raised neutrophils with a white-blood-cell count of 14509 cells/mm^3^ and raised C-reactive protein (CRP) levels. A urine sample showed hematuria and proteinuria. A clinical diagnosis of Henoch-Schönlein Purpura (HSP) was made. The classic tetrad of symptoms in HSP includes palpable purpura (small red or purple spots on the skin), arthritis or joint pain, abdominal pain, and renal involvement. In adults, arthritis and joint pain may be more pronounced and persistent compared to children. The rash appears as small red or purple spots on the skin and is often distributed symmetrically on the lower extremities, especially around the ankles and buttocks. It may also involve the arms, thighs, and occasionally the trunk. The rash is palpable, meaning it can be felt by running a finger over the affected area. The skin may feel slightly raised or bumpy due to inflammation and the presence of small blood vessels affected by vasculitis. Diagnosis of HSP in adults is based on clinical presentation, physical examination, laboratory tests (including inflammatory markers, urinalysis, and renal function tests), and sometimes, skin biopsy. In severe cases, management typically involves supportive care, pain control, anti-inflammatory medications (such as NSAIDs), and corticosteroids. Our patient was managed intravenous fluids, diclofenac sodium for arthralgia, and a short course of 5 days of intravenous hydrocortisone. The patient improved symptomatically and the rash resolved over 3 weeks.

**Figure 1:**
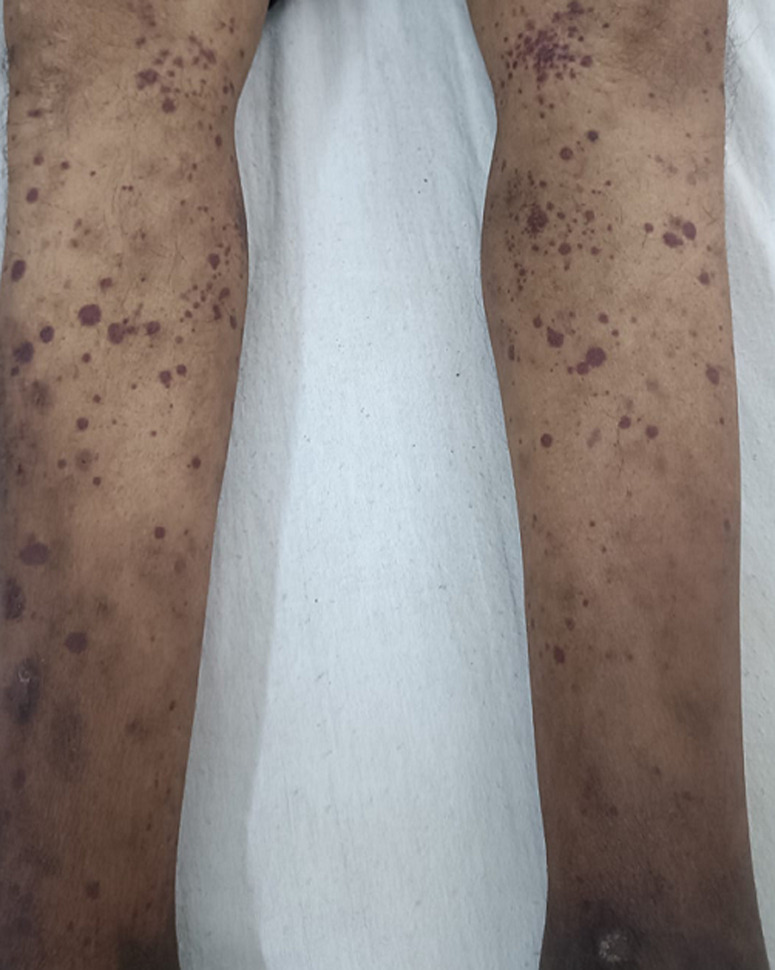
Henoch-Schönlein Purpura (HSP) rash on lower limbs

